# Evaluating the specialist palliative care clinical nurse specialist role in an acute hospital setting: a mixed methods sequential explanatory study

**DOI:** 10.1186/s12904-021-00834-y

**Published:** 2021-09-03

**Authors:** Michael Connolly, Mary Ryder, Kate Frazer, Eileen Furlong, Teresa Plazo Escribano, Philip Larkin, Eileen Carruthers, Eileen McGuigan

**Affiliations:** 1 UCD School of Nursing, Midwifery &Health Systems, University College Dublin, Dublin, Ireland; 2 Palliative and Supportive Care Service, Lausanne University Hospital and University of Lausanne, Lausanne, Switzerland; 3 Regional Specialist Palliative Care Services, Louth, Meath, Cavan, and Monaghan, Drogheda, Ireland

**Keywords:** Specialist palliative care, Clinical nurse specialist, Acute hospital, Core competencies

## Abstract

**Background:**

Special palliative care is provided in a range of settings including a patient’s home (their primary place of dwelling), a hospice in-patient unit, or an acute hospital. The aim of the study was to evaluate the role of the specialist in palliative care clinical nurse specialist (SPC CNS) role in an acute hospital setting.

**Methods:**

This study was conducted using a mixed methods sequential explanatory approach in two phases; phase 1 involved completion of a study questionnaire (*n* = 121) and phase 2 involved part-taking in a focus group (*n* = 6) or individual interview (*n* = 4).

**Results:**

Phase 1 results indicated that respondents held positive attitudes towards the Specialist Palliative Care Clinical Nurses Specialist (SPC CNS) in relation to clinical care, education and patient advocacy. Phase 2 qualitative findings identified the importance of the role in terms of symptom management, education and support.

**Conclusions:**

This study provides an evaluation of a SPC CNS role since it was established in an acute hospital setting. The evidence indicates that there is a varied understanding of the role of the SPC CNS. The role was seen as an important one particularly in terms of referrals to and support provided by the SPC CNS, as well as recognition of the importance of the role is providing ongoing education to staff.

## Background

The World Health Organisation defines Palliative Care as:

an approach that improves the quality of life of patients and their families facing the problem associated with life-threatening illness, through the prevention and relief of suffering by means of early identification and impeccable assessment and treatment of pain and other problems, physical, psychosocial and spiritual [1].

In the Republic of Ireland there are three levels to providing palliative care; the palliative care approach, general palliative care and specialist palliative care depending on practitioner’s skills and the patient’s needs. This study focussed on the establishment of a specialist palliative care nursing service in one acute hospital. Specialist Palliative Care (SPC) is provided in a range of care settings including a patient’s home (their primary place of dwelling), an acute hospital, or a hospice in-patient unit. In Ireland SPC is a consultant-led service with nurses providing direct and largely specialist care. Therefore, specialist palliative care nurses often work independently, albeit as part of a larger multi-disciplinary team, and are seen as a reference point for clinical advice and guidance, planning care, managing discharge planning and family support and deal with the breadth of physical, psychosocial and spiritual concerns commonly seen in the context of palliative and end-of-life care.

Specialist nursing roles emerged in Ireland during the millennium following industrial action calling for clinical career progression opportunities for the profession [2]. As an outcome of this report, The National Council for Professional Development of Nursing and Midwifery outlined two distinct advanced practice nursing roles, Clinical Nurse Specialist (CNS) and Advanced Nurse Practitioner (ANP) [3]. Consistent with the more recent international definition, the CNS role is designed to improve quality of patient care through the application of specialist knowledge and skills to a defined specialist patient population with established diagnoses [4]. In a clinical focused role the CNS has the authority to amend prescribed patient treatments within agreed protocols and guidelines, audit practice and evaluate improvements to the quality of patient care [3]. While the CNS role in Ireland has defined requirements and standards for educational preparation, it is not supported by a regulatory framework with practice standards [5].

Internationally, the CNS has evolved over time to meet clinical gaps in patient care. Thus, at times it can be both difficult to define the CNS role and clearly articulate their value to the care of patients and families. The CNS is in a unique position that allows them to enhance patient care through their involvement in general practice, counselling of staff, education and research, and providing clinical leadership through their consultative role [6–8]. The CNS is often in contact with patients, their significant others, other nurses and members of the multidisciplinary team. The CNS is also in regular contact with nursing and hospital management and can see the difficulties in providing nursing care using the resources available in daily practice. That said, the CNS is also uniquely positioned to prioritize the necessary interventions and initiate solutions to problems at various levels [6–12].

Relationships between the CNS and general nurses working in an acute care environment, are important to optimize care, so mutual role expectations and conditions are established and maintained so that generalist nurses are empowered and are not de-skilled [13, 14]. It is important to note that familiarity with the CNS role and their contribution to care does not guard against the potential for such dis-empowerment and loss of skills [15]. Evidence suggests that having clear role definitions and objectives that are clearly communicated to relevant staff when introducing new roles, is fundamental to preventing role ambiguity and the likelihood of negative responses [6, 16].

The clinical nurse specialist role in palliative care has also evolved and is evident community palliative care teams and acute hospital services [10, 17, 18]. While this expansion in specialist palliative care nursing has been evident, there is a dearth of evidence on the impact of these roles on services, with little serious consideration as to how their role effectiveness might be assessed [6, 14, 19–24].

To date only a small number of studies have been undertaken specifically focussing on the SPC CNS. Jack et al. [18] evaluated the impact of the palliative care clinical nurse specialist with doctors and nurses (*N* = 31), within an acute hospital setting. Using semi-structured interviews to elicit opinion, they found that the CNS was beneficial to both medical and nursing staff. Skilbeck and Payne [25] echoed these findings but were also at pains to point out the significant level of emotional care and support that the CNS provides to the patient and their family. Focussing on the evolving role of the community CNS in palliative care, Husband [26] reaffirmed previous findings regarding the continually evolving role of the CNS, with an ever-widening remit including clinical, educational, research and other responsibilities. Husband [26] found that CNS’s easily recognised the importance of managing both clinical and educational components of their role but found it hard to balance the other demands. An important factor to support continued development for the CNS were clearly defined criteria for both the role and their responsibilities. A Northern Ireland based study of community CNS’s in Palliative Care demonstrated that participants found their CNS role stressful and that both organisational and individual culture influenced both their professional and personal development [27, 28], while Firm et al. [28] in their narrative synthesis concluded that more integration of specialist palliative care services and timely sharing of information would further enhance the relationship of the SPC CNS with the hospital community.

In Ireland, Cowman et al. [29] undertook a study to evaluate the role of the CNS in cancer care. Findings indicated that the CNS was highly educated and perceived to be an active member of the MDT. However, the findings also demonstrated that the research role needed development.

## Methods

The aim of the study was to evaluate knowledge and perceptions of the role of the specialist palliative care clinical nurse specialist (SPC CNS) in an acute hospital setting.

This study was conducted using a mixed methods sequential explanatory approach in two phases [29–33]; an initial quantitative phase followed by an qualitative phase to explain the initial quantitative results [29]. In Phase 1 an anonymised survey of key stakeholders, hospital managers, nurses, nurse managers, doctors and allied health professionals in the hospital was conducted to identify their knowledge of the SPC CNS role in providing palliative care. The survey instrument was adapted from one used previously by Cowman et al. [29]. The paper-based questionnaire, with a sealed envelope, was distributed to the hospital by a gatekeeper who visited the various departments to encourage staff engagement with the research study and the survey and collected completed questionnaires. The gatekeeper works on the palliative care team across the geographical sector and while known to some staff at the hospital is not directly employed there. Data were analysed using IBM SPSS Statistics 24 and descriptive statistics are reported.

Phase two, aimed to explore issues gleaned from the quantitative phase of data collection providing an opportunity for a deeper understanding of SPC CNS role and the impact of the role in the study site. Focus groups were conducted with multi-professional staff who had completed the study questionnaire and expressed a willingness in response to a question to participate in a focus group. Potential participants were selected randomly from, 20 individuals who had provided contact details on their completed questionnaire. Additionally key stakeholders were invited to participate in an individual interview to evaluate their understanding of the SPC CNS role and the service provided.

Two focus group interviews, with a total of six participants and four individual interviews were conducted. The focus group interviews were scheduled to take place in a designated meeting room at the hospital. On each of the planned days for the focus group interviews, pressure of work meant those who had originally agreed to attend were unable to do so resulting in a lower than expected number of attendees at the focus group interviews. The duration of interviews was between 20–45 min. The focus groups were undertaken in the hospital where the study was located. To facilitate individual interviews, three were conducted by phone contact, with the fourth completed face to face. An interview schedule was guided by questions from the survey and was informed by the initial quantitative analysis and used for both the focus group and individual interviews. Each interview was audio recorded and all were transcribed verbatim. Qualitative data were analysed using MAXQDA 11. Thematic analysis of the data enabled the identification of patterns and themes [32].

## Results

### Phase 1

A total of 200 questionnaires were distributed to nurses, physicians, and health and social care professionals throughout the study site. 121 questionnaires were returned yielding a response rate of 61%.

Table 1 presents a profile of respondents’ roles with the majority holding nursing roles. Similarly, eleven discrete clinical areas are represented indicating broad participation in the survey. Most respondents had over 11 years of clinical experience; only a minority (14%) had less than 5 years of clinical experience in their role (Table 2).

**Table 1:** Profile of current role and location

	***Frequency (n* = *121)***	***Percentage***
***Role***		
Staff Nurse	64	52.9
Clinical Nurse Manager (1 or 2)	14	11.6
Clinical Nurse Specialist	13	10.7
Senior Nursing Manager (CNM 111 or higher grade)	1	0.8
Physiotherapist	10	8.3
Doctor (Medical and Surgical)	6	5.0
Health and Social Care Professionals (e.g. Dietician, Occupational Therapist, Psychologist, Speech and Language Therapist, Social Worker	3	2.5
Other (e.g. specific professional role in hospital identified)	10	8.2
***Total***	***121***	***100.0***
***Location***		
Acute Medical Assessment Unit	8	6.6
Acute Surgical	6	5.0
Coronary Care Unit	3	2.5
Day Ward	3	2.5
Emergency Department	4	3.3
Intensive Care Unit	7	5.8
Medical Ward	42	34.7
Obstetrics and Gynaecology	2	1.7
Out-patients Department	15	12.4
Short Stay Unit	4	3.3
Surgical Ward	1	.8
Other (specify)	26	21.5
***Total***	***121***	***100.0***

**Table 2:** Duration of clinical experience

**Years**	**Frequency**	**Percentage**
< 5 years	17	14.0
5 to 10 years	23	19.0
11 to 20 years	36	29.8
> 20 years	45	37.2
***Total***	***121***	***100.0***

The majority of respondents were aware that SPC CNS was employed in the hospital (99.2%; *n* = 120) and data identified that over fifty percent of respondents (53.7%; *n* = 66) had referred a patient to the SPC CNS in the past 12 months.

Data showed that the primary reason for referral was symptom management (71% *n* = 86) with a sizable minority selecting bereavement support (11%; *n* = 14) (Table 3). The data indicated that most referrals to the SPC CNS, across all categories were received from nurses with some referrals from other health and social care professional.

**Table 3:** Reason for advice sought from clinical nurse specialist ranked highest

**Reason for advice**	**Frequency (*N* = 96)**	**Percentage**^**a**^
Symptom Management	86	71.1
Communicating bad news	21	17.4
Bereavement support	14	11.6
Education of staff	19	15.7
Staff support	22	9.1
Discharge planning	55	45.4
Other	2	1.6

^a^Respondents selected more than one response

Overall respondents held positive attitudes towards the SPC CNS role especially in relation to clinical care, education and patient advocacy (Table 4). While a majority (78%) indicated that audit and research were core activities of the role.

**Table 4:** Attitudes to clinical nurse specialist role

	**Strongly agree %/n**	**Agree %/n**	**Somewhat agree %/n**	**Disagree %/n**	**Strongly disagree %/n**
The CNS in Palliative care has a very important clinical role (*n* = 121)	90.1 (109)	6.6(8)	1.7 (2)	0.0	1.7 (2)
The CNS in Palliative Care always plays an integral role in patient education (*n* = 121)	86.0 (104)	10.7 (13)	1.7 (2)	0.0	1.7 (2)
The CNS in Palliative Care always plays an integral role in staff training and education (*n* = 121)	68.6 (83)	20.7 (25)	6.6 (8)	1.7 (2)	2.5 (3)
The CNS in Palliative Care is a patient advocate (*n* = 121)	82.6 (100)	14.0 (17)	2.5 (3)	0.0	0.8 (1)
Audit and research are core activities of the role of CNS in palliative Care (*n* = 120)	40.5 (49)	38.0 (46)	14.9 (18)	4.1 (5)	1.7 (2)

Respondents were asked to rank the core competences for SPC CNS. Almost two thirds of respondents ranked clinical competency role as their central core competence (65.3%; *n* = 79). One fifth of respondents selected advocacy as their central core competency (20.7%; *n* = 25) (Table 5).

**Table 5:** Core competence ranked 1^st^ by professional role

	**Clinical %/n**	**Advocacy %/n**	**Consultation %/n**	**Education %/n**	**Audit/Research %/n**	**Total (n)**
Dietician	50.0(1)	0	50.0(1)	0	0	2
Staff Nurse	62.5(40)	26.6(17)	7.8(5)	1.6(1)	1.6(1)	64
Clinical Nurse Manager I or II	64.3(9)	14.3(2)	14.3(2)	0	7.1(1)	14
Clinical Nurse Specialist	69.2(9)	7.7(1)	15.4(2)	7.7(1)	0	13
Senior Nurse Manager (CNMIII; ADON; DON)	100.0(1)	0	0	0	0	1
Medicine – Physician	33.3(1)	33.3(1)	33.3(1)	0	0	3
Medicine – Surgeon	100.0 (3)	0	0	0	0	3
Physiotherapist	80.0(8)	10(1)	10(1)	0	0	10
Occupational Therapist	100(1)	0	0	0	0	1
Other	60.0(6)	30.0(3)	0	0	10(1)	10
**Total**	65.3(79)	20.7(25)	9.9(12)	1.7(2)	2.5(3)	121

Finally, one third of respondents identified they had education on palliative care provided by the SPC CNS in the past year (33.1%; *n* = 40). It is of note that few respondents had any collaboration with the SPC CNS concerning audit (5%; *n* = 6), research (2.5%; *n* = 3) or quality improvement (7.4%; *n* = 9).

### Phase two findings

Findings from the qualitative data are presented under the themes and sub-themes that emerged. The main themes emerging were: The role of the specialist palliative care clinical nurse specialist (SPC CNS); Things being done well, and Competence.

### The role of the specialist palliative care clinical nurse specialist

Focus group and individual interview participants were asked to reflect on their understanding of the role of the specialist palliative care clinical nurse specialist (SPC CNS). A varied understanding of the role that the SPC CNS for some respondents. The role was described as educative and supportive, ‘about advocacy’ – for the patient and their family, a sense of continuity in care, and was a resource to support the care of patients with complex needs:

I believe the role is that of, a supportive one, I don’t see it as that of hands on care. But I see it very much as being very involved in the patient and family’s care and journey. But through information, through support, through support of the patient and family but support of other staff members, whether they be nursing, qualified nurses, whether they be health care assistants, whether they be junior doctors or indeed whether they be other consultants from other teams. (P5)

My understanding of the role is one of advocacy number one for the patients that we’re looking after. And also helping out with the family, the palliative care patient, their family and anybody special to them. (P6)

While differences in focus on the role of the SPC CNS was evident, there was general agreement in the context of specific role components. For both focus group and individual interview participants, symptom management was seen as a key area where the SPC CNS provided guidance and expert advice:

… from our perspective we would link in with the palliative services very much for symptom control … So very much you know working side by side with palliative care has really been an advantage to some of our clients. (P1)

Helping with symptom management, all aspects of their care including psychosocial, spiritual, physical etc. (P5)

A key aspect of the role of a clinical nurse specialist is the provision of education to both patients and fellow clinicians about a particular condition and how best to manage it. Clinical Nurse Specialists can draw on their expert knowledge and clinical experience to provide this education. Education played an important and central role for the SPC CNS, with participants seeing this role as the most important in the context of support:

I suppose education is paramount, assisting them to assist the patient so giving them the tools they need to look after the patient in the absence of the team. The tools then need to I suppose identify symptoms and how to treat them effectively … So there’s a huge emphasis or there should be a huge emphasis on education. (P5)

By supporting, encouraging, doing education … to really support the best outcomes for the patients proceeding whether it would be general palliative care or specialist. (P6)

While education was seen to be important there was concern that current provision of education sessions was ad-hoc and needed to be provided in a more formal and structured way:

I suppose the feeling would be from my personal perspective is that education is at present very ad hoc and at the bed side and in the treatment room or whatever rather than formal education which I feel is, can only help the patient in the longer run, you know. So I feel that you know the role could have a much more active role in teaching and formal education of staff, both medical, nursing and multidisciplinary with regards to symptom management especially of the patients within that cohort. (P5)

The supportive role of the SPC CNS in supporting staff, particularly in the context of the many issues that arise when managing patients with complex needs and patients at end-of-life emerged.

… the support that they give the staff and also the family and maybe opening up that conversation and supporting them in that conversation. (P1)

… support to ward staff with regards to the care of patients as they come towards end of life. (P2)

A key component of this support focussed on discharge planning particularly when arranging a rapid discharge, to facilitate a person’s wish to die at home.

… dealing with maybe planning discharge home and support in the community and all that sort of thing. (P1)

I know previously … you know you would dread the thought of a palliative patient going home because it was such a complex discharge (P3).

### Things being done well

There was some agreement among both focus group and individual interview participants that the SPC CNS service has been a welcome addition to the hospital. The role of SPC CNS was viewed as an important in the context of the multidisciplinary team, the provision of person-centred care and strongly advocating for the patient and their family:

I think you know that MDT approach that they have is working well. (P5)

… you know they will advocate for the needs or the you know the complexity that is going on with the family and what might be required or needed for them… it’s safe to say its interacting with the patient and their families, works extremely well. (P6)

Participants were asked to consider what if any changes they might consider making to the current service to make improvements or assist the ongoing development.

For some it was structural changes that were articulated including the provision of a dedicated workspace for the SPC CNS service. Other considerations included the inclusion of dedicated palliative care beds for admission and management of patient care.

The need for greater clarity on the SPC CNS service role and the expectations of the service provided was articulated by a number of respondents:

I think there’s real confusion and lack of clarity about role expectation. I don’t think that’s anybody’s fault. I think there’s a clear vision but the understanding is lacking … There is a … disconnect in how the clinical nurse specialist, nurse managers and the consultant see the service. And indeed it’s also very different to what other clinical nurse specialists might believe. So I think where change is needed is an agreed vision of some sort for that role. (P5)

I suppose the roles of the nurse…has never been clearly defined within the setting in which SPC CNS role is. So from the get go ... it has been massively clinical and I even dare to say massively medical in its approach. (P6)

What is evident is that a full understanding of the role of the SPC CNS is lacking, particularly from consultant physicians who are the lead clinicians of the multidisciplinary team. Importantly the role of the SPC CNS to be seen as an autonomous practitioner needs to be asserted:

I believe as well that … consultant colleagues whilst they value and I know that they value the work of [the SPC CNS service] ... I’m not convinced they completely understand the role of the clinical nurse specialist. (P5)

So it’s very much led by a consultant and I suppose the role of the CNS within that team is much less of an autonomous role than any other CNS within the hospital. Most of the CNSs within the hospital and that would be the case throughout the country work autonomously within the speciality in which, they speak to and they consult with different members of the medical team, not specifically one. (P6)

But I think there needs to be what is the word not be handmaidens as much as more autonomous practitioners. (P7)

### Competence

Core competencies for the Clinical Nurse Specialist roles are defined by the NCNM [6] clinical, advocacy, consultation, education, audit and research and participants were asked to consider the competencies and rate them as either of most or least important.

Although responses varied, in general the clinical role of the SPC CNS was of significant importance, as well as the advocacy role:

And I think it’s interesting that you say clinical and advocacy and they are closely linked but you know you’re very experienced, palliative care nurse is but sometimes you know if you do see a disjoint between them, you can see that they’re needed together but not everyone has the expertise that people like you have, do you know what I’m saying in both being clinical. (P1)

I would say probably clinically, for the patient to know how the patient is, how the family is coping and I know, I mean the research and audit has to be done but at the end of the day the consultant is driving the service, like regards to what the latest research is unless the consultant is up with it and doing it, there’s not much point in me saying well this is the latest. (P3)

That said participants identified that all competencies were of equal importance to the role and service and no one competence should be deemed more important than any other:

Like the clinical the advocacy piece the education piece I can’t remember is it audit and research and their consultant role I see them all as equally important but I don’t see the role as you know I worry that the role doesn’t have that autonomy that it should have. (P7)

The vast majority of participants indicated that audit and research were of least importance to the role of the SPC CNS. However, variance in opinions were voiced. For some research was viewed as a consultant led activity, indicating that the SPC CNS would have a limited role in research:

I mean the research and audit has to be done but at the end of the day the consultant is driving the service, like regards to what the latest research is unless the consultant is up with it and doing it, there’s not much point in me saying well this is the latest. (P3)

Audit and research although perceived as important, some felt that this involved a lot of work with no time to complete it due to the other demands of the role:

I suppose auditing, you know what I mean … so much time on the ward, with the patients and all that, to have to do stuff like paper work and audit is taking [you] away from all of that. So I know it’s important and I do recognise the importance of the auditing but … god you know I’m sure [you’d] be here all hours trying to fit that part in, you know the paperwork part of it. (P3)

Finally, a varying view of competence saw it as primarily focussed on physical symptom management, with some focus on psychological care, but a total neglect of spiritual care:

You see all this competencies, in my opinion, in practice they melt down to physical symptom management. And there is maybe a little bit of psychological merit. But the spirit dimension is completely neglected. (P8)

Going further this participant tended to see each of the core competencies in isolation:

I think, to be honest, the difficulty … to deliver in all these competencies. And the result is that … doesn’t deliver in any of the competencies, in a consistent way. If you put too much demand, you only can frustrate the person who does it. And the people who get the service. (P8)

## Discussion

This study provides an evaluation of a SPC CNS role since it was established in an acute hospital setting, 18 months prior to the commencement of the study. While the evidence presents a varied understanding of the role of the SPC CNS, its importance was strongly reinforced in terms of referrals and support provided from both quantitative and qualitative phases.

Principal focus for referrals to the SPC CNS were referrals for symptom management, communicating bad news and bereavement support (Table 3). Overall respondents held positive attitudes towards the SPC CNS in relation to clinical care, education and patient advocacy. A minority of respondents (< 10%) did not value audit and research as core activities of the role.

Qualitative findings identified the importance of the role in terms of symptom management, education and support. The role was viewed primarily as end-of-life care and for management of symptoms. There are opportunities for further development of the role for the management of chronic illness. The role was valued highly and seen as important in the context of the multidisciplinary team, and the provision of person-centred care as an advocate for patients and families.

The core competences identified in both the survey both focus group and individual interviews emphasise the importance for symptom management and discharge planning. It is not surprising that most referrals and supports were sought by nurses as they are the majority of respondents in the study and comprise the majority of professionals working in the hospital.

Attitudes to the SPC CNS role were highly positive for all areas relating to their role from both quantitative and qualitative data. Their clinical role, integration with patient and staff education were all recognised by a sizable majority of respondents and within qualitative findings with their clinical expertise in knowledge, education and management of symptoms were all articulated and acknowledged by participants.

Whilst the core competences for the Clinical Nurse Specialist role are as clinical, advocacy, consultation, education, audit and research [3, 6]; findings from this evaluation provides ranking of competencies in relation to the role. Clinical competency and advocacy were ranked highest and these competencies as most important. However, data from the focus groups and individual interviews present challenges. This is particularly true in respect of the perception of a significant lack of understanding of the role of the CNS by consultant colleagues who are ultimately responsible for leading clinical services. This is reflective of research with similar findings and is consistent with the understanding of many specialist and advanced practice nursing roles internationally [34, 35]. This lack of understanding will need to be addressed as the SPC CNS services continues to be provided and is likely to expand over time.

While competencies were valued equally, education was seen as key, and it is notable that one third of respondents attended a formal education session on palliative care during the previous 12 months.

While the role was seen as supportive, the structural barriers of lack of dedicated beds for admitting patients emerged as a challenge. Role ambiguity exists and is not surprising given the early nature of the evaluation and provides a basis for further development of the role of the SPC CNS. Such ambiguity can be addressed by ensuring that a clear role description for the SPC CNS is developed and implemented. It is also important that all members of the multidisciplinary team are provided with education about the SPC CNS role [13, 28, 35, 36]. There is clearly an appetite to see the SPC CNS role further developed in the future to address patient and family needs and support staff and this is clear from both the survey and qualitative findings.

## Limitations

Although the response rate to the questionnaire was reasonable (61% response rate), with most respondents being nurses. An increased response from other health and social care professionals would have added to the results. Despite encouragement and planning the number of individuals who were available to attend focus groups was fewer than anticipated – this could in part be due to the pressure of working conditions in an acute health care environment which limits time available to engage with research during working hours. A significant limitation was the lack of a patient’s voice in the evaluation. This omission was due to time limitations of the evaluation. It is essential to prioritise the inclusion of patients’ voices in the development of the role and future evaluations of the service.

## Conclusions

No definition of the SPC CNS role exists in practice and this vacuum creates differences in expectations and perceptions of care provision.

It is important for service provision and future development that the role and responsibilities of the SPC CNS should be clearly articulated to ensure the role is fully understood by all health and social care professionals. The SPC CNS service should be fully embedded in the hospital including the provision of infrastructure to support the clinical, advocacy, consultation, education, audit and research work of the SPC CNS. As the role continues to develop it is important to ensure that key priorities for service, personal and professional development over the next 12 to 18 months are discussed with the Palliative Care team and where appropriate identify developments that could be led by the SPC CNS. This discussion must also include consideration about how engagement in audit and research should be facilitated for the SPC CNS.

## Data Availability

The datasets used and/or analysed during the current study are available from the corresponding author on reasonable request.
